# Effect of high-dose intravenous vitamin C on organ support-free days within the first 14 days in severe acute pancreatitis patients: a multicenter, randomized, double-blind, placebo-controlled clinical trial protocol

**DOI:** 10.3389/fnut.2026.1850105

**Published:** 2026-08-06

**Authors:** Bing Zhao, Xianxian Yu, Haibo Zhang, Meiling Yu, Wenwu Sun, Fengying Zhao, Hua Huang, Menglong Song, Shu Wang, Wenjie Ma, Meihua Shen, Shuyuan Zhao, Yihui Wang, Menglu Gui, Dan Xu, Mengqi Gao, Huiqiu Sheng, Li Ma, Weisong Jiang, Ning Ning, Silei Sun, Yang Li, Qionghua Hu, Xiaojiao Liu, Ping Zhou, Yang Tao, Jun Huang, Yue Zeng, Meitang Wang, Ruilan Wang, Enqiang Mao

**Affiliations:** 1 Department of Emergency, Ruijin Hospital, Shanghai Jiao Tong University School of Medicine, Shanghai, China; 2 Department of Emergency, Medical Center of Trauma and War Injury, Daping Hospital, Army Medical University, State Key Laboratory of Trauma and Chemical Poisoning, Chongqing, China; 3 Department of Critical Care Unit, The Second People's Hospital of Chengdu, Sichuan University, Chengdu, Sichuan, China; 4 Department of Critical Care Unit, Guanghan People's Hospital, Deyang, Sichuan, China; 5 Department of Emergency Intensive Care Unit, Sichuan Provincial People's Hospital, Chengdu, Sichuan, China; 6 Department of Intensive Care Medicine, Chongqing University Central Hospital (Chongqing Emergency Medical Center), Chongqing, China; 7 Department of Emergency, Changhai Hospital, Naval Medical University, Shanghai, China; 8 National Center for Translational Medicine (Shanghai) SHU Branch, Shanghai University, Shanghai, China; 9 Department of Critical Care Unit, Shanghai Provincial Corps Hospital, Chinese People’s Armed Police Forces, Shanghai, China; 10 Department of Cardiovascular Medicine, State Key Laboratory of Medical Genomics, Shanghai Key Laboratory of Hypertension, Shanghai Institute of Hypertension, Ruijin Hospital, Shanghai Jiao Tong University School of Medicine, Shanghai, China; 11 Department of Gastroenterology, Shanghai General Hospital, Shanghai Jiao Tong University School of Medicine, Shanghai, China; 12 Department of Emergency and Critical Care Unit, Shanghai General Hospital, Shanghai Jiao Tong University School of Medicine, Shanghai, China

**Keywords:** high-dose intravenous vitamin C, intensive care unit, multicenter, randomized controlled trial, severe acute pancreatitis

## Abstract

**Background:**

The global incidence of severe acute pancreatitis (SAP) is increasing, yet mortality rates remain persistently high. Novel therapeutic approaches are urgently needed to improve outcomes. The aim of this study is to evaluate the efficacy of high-dose intravenous vitamin C in improving organ support-free days within the first 14 days and other clinical outcomes in patients with SAP.

**Method:**

This is a prospective, randomized, double-blind, multicenter clinical trial. Participants will be stratified and randomly assigned to either the HDIVC group or the control group. Data will be analyzed using SAS 9.4 with two-sided tests and a significance threshold of *p* < 0.05. This study will be conduct at eight hospitals in China, including Shanghai Jiao Tong University School of Medicine Affiliated Ruijin Hospital, Shanghai General Hospital, Changhai Hospital, Sichuan Provincial People’s Hospital, Chongqing Daping Hospital, The Second People’s Hospital of Chengdu, Guanghan People’s Hospital and Chongqing Emergency Medical Center. Eligible patients are adults (age ≥ 18 years) diagnosed with severe acute pancreatitis according to 2012 Atlanta classification within 7 days of onset. HDIVC (500 mg/kg, 2 g/h) or placebo (normal saline) will be administered via central vein for 7 days. The primary outcome is organ support-free days within the first 14 days. Secondary outcomes include ICU all-cause mortality, changes in inflammatory biomarkers, and organ failure scores.

**Discussion:**

If proven effective, the results of this trial could establish high-dose intravenous vitamin C as a cost-effective adjunctive therapy to improve survival in patients with severe acute pancreatitis and potentially inform future clinical practice guidelines.

**Clinical trial registration:**

ClinicalTrials.gov, identifier NCT06897384.

## Introduction

Severe acute pancreatitis (SAP) is the most life-threatening form of acute pancreatitis (AP), and its incidence is rising globally (1). In Western countries, annual incidence runs from 13 to 45 per 100,000. In China, rates climbed from 0.19 to 0.71% over the past two decades. Mortality still sits between 13 and 35% despite better management (2).

Antioxidant therapy is important in SAP management. During early disease, oxidative stress from infection and uncontrolled reactive oxygen species (ROS) release triggers systemic inflammatory response syndrome (SIRS), which can progress to multiple organ dysfunction syndrome (MODS) (3). Therefore, timely elimination of excessive ROS is considered as a critical therapeutic target for improving clinical outcomes.

Vitamin C, a potent plasma antioxidant, effectively neutralizes ROS (3). Preclinical and clinical studies have demonstrated its anti-inflammatory properties and endothelial protective effects (4–6). Although oral administration is common (7), it fails to achieve therapeutic plasma concentrations in critically ill patients. Because critical illness drives rapid ROS overproduction and impairs gut absorption, oral dosing raises plasma levels to only around 150 μmol/L, well below what is needed to affect oxidative stress (8). Recently, high-dose intravenous vitamin C (HDIVC) has emerged as a promising intervention in critical care therapy. Growing evidence supports its potential efficacy in SAP, acute respiratory distress syndrome (ARDS), and severe coronavirus disease 2019 (COVID-19) (9).

HDIVC exerts multifaceted therapeutic effects through four pathways (10). Primarily, it functions as a potent antioxidant by directly eliminating ROS and reactive nitrogen species (RNS) while modulating enzymatic ROS through NADPH oxidase inhibition and other endogenous antioxidant system activation (11, 12). Second, as a critical cofactor in catecholamine biosynthesis, it bolsters hemodynamic stability through epinephrine and dopamine production. It simultaneously promotes vasopressin synthesis, raising blood pressure via R1 receptors, maintaining intravascular volume via R2 receptors, and increasing glucocorticoid synthesis via R3 receptor-driven ACTH release (13). Third, it contributes to immune defense by promoting lymphocyte proliferation, neutrophil chemotaxis and bacterial killing efficacy (13, 14). Finally, it decreases inflammatory activity through NF-kB pathway inhibition. These synergistic mechanisms position HDIVC as a promising therapeutic intervention for critical illness.

In our preclinical work, vitamin C supplementation lowered inflammatory markers, mortality, and pancreatic necrosis in SAP mice (15, 16). Whether this carries over to humans is unclear. Existing clinical data are inconsistent and come from small samples, insufficient dosing, or cohorts weighted toward mild disease (17). From September 2019 to December 2023, we conducted a single-center, double-blind, randomized, placebo-controlled trial (ChiCTR1900022022) investigating HDIVC (200 mg/kg/24 h, 1 g/h, for 7 days) (6). The primary outcome of 28-day mortality did not differ significantly between treatment groups in the overall cohort (*n* = 212). However, prespecified subgroup analysis revealed that MSAP patients receiving HDIVC demonstrated significant reductions in C-reactive protein levels and improved fluid balance. These findings suggest HDIVC may attenuate inflammatory responses in MSAP, though its therapeutic value in SAP requires confirmation through adequately powered multicenter randomized controlled trials. Extensive preclinical investigations over the past few decades have established the therapeutic potential of HDIVC in SAP, with mechanistic understanding progressively refined through cellular, animal, and early-phase human studies. While these foundational studies show biological plausibility, current clinical evidence remains limited to underpowered single-center trials, precluding definitive conclusions about therapeutic efficacy. To address this critical evidence gap, we propose a multicenter, prospective, randomized, double-blind, placebo-controlled trial evaluating early HDIVC administration (500 mg/kg/day for 7 days, 2 g/h) in SAP patients. The primary outcome is organ support-free days (OSFD) within the first 14 days. Secondary outcomes include ICU all-cause mortality, inflammatory biomarker, organ failure scores and fluid retention. This study aims to determine whether HDIVC represents a clinically meaningful, cost-effective therapeutic intervention capable of improving outcomes in this high-mortality condition, with potential to inform future guideline recommendations.

## Method and analysis

### Study design

**Figure 1:**
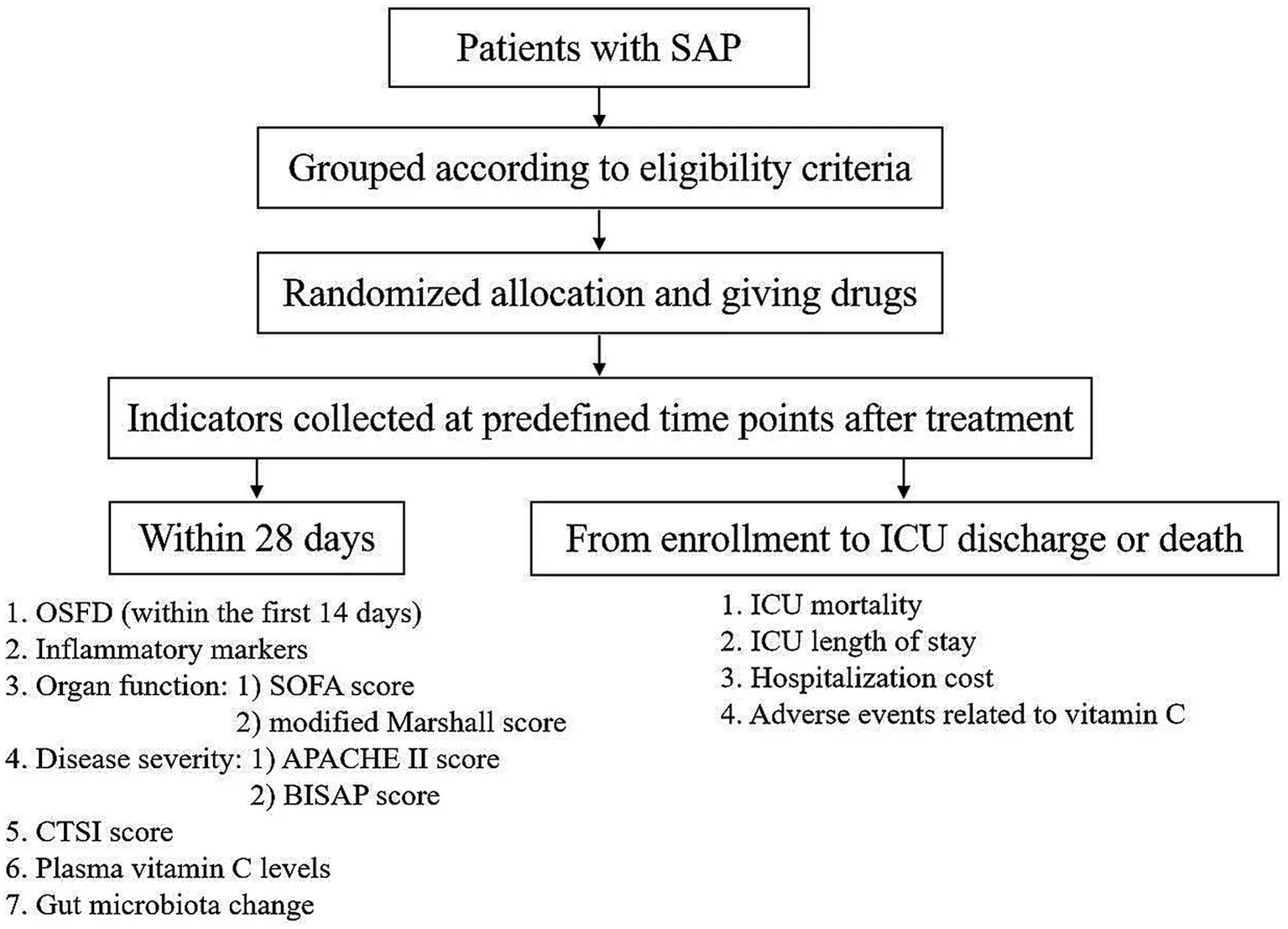
Flowchart of participants. This diagram shows the trial processes and outcome assessment. SAP, severe acute pancreatitis; ICU, intensive care unit; OSFD, organ support-free days; SOFA, sequential organ failure assessment; APACHE II, acute physiology and chronic health evaluation II; BISAP, bedside index for severity in acute pancreatitis; CTSI, CT severity index.

This prospective, randomized, double-blind, multicenter trial will investigate whether early intravenous HDIVC reduces OSFD within the first 14 days in SAP patients. We will enroll 238 participants (119 per group) from eight hospitals across China, with block randomization allocating patients to either HDIVC or control groups. Serial assessments of organ function, disease severity scores, and mortality will be evaluated at predefined time points before and after treatment to determine therapeutic efficacy. The evaluation and examination will be continued from randomization to 180 days or death. Figure 1 shows the flow chart of the study. This study protocol is written according to the Standard Protocol Items for Randomization Trials (SPIRIT) statement (18).

### Eligibility criteria

#### Inclusion criteria

Participants must meet the following criteria:

1. Age ≥ 18 years;
2. Time from onset to enrollment is less than 7 days;
3. Meets the diagnostic criteria for SAP per the 2012 Atlanta Guidelines, defined by persistent organ failure (modified Marshall score of 2 or higher lasting more than 48 h). Confirmation of the 48-h persistence must be completed within 7 days of disease onset.

#### Exclusion criteria

Participants who meet any of the following criteria will be excluded from the study:

1. SAP caused by tumors or ERCP;
2. Pregnancy or breastfeeding;
3. Allergy to vitamin C;
4. Use of other experimental drugs during the study period;
5. Pre-existing chronic organ failure affecting the heart, liver, lung, or kidney prior to admission, defined as: chronic cardiovascular dysfunction requiring long-term mechanical hemodynamic support or inotropic drugs; chronic obstructive pulmonary disease requiring home oxygen therapy; chronic liver failure classified as Child-Pugh grade C; chronic kidney disease defined by eGFR less than 60 mL/min/1.73 m^2^ (CKD-EPI 2021). Baseline creatinine is based on stable outpatient values from the past 6 months; if unavailable, exclusion relies on a documented history of chronic kidney disease rather than an acute admission creatinine.
6. Immunosuppressive states, including malignant tumors, post-transplant conditions, long-term use of immunosuppressor (for at least 1 month before enrollment), or AIDS;
7. Refuse to sign the informed consent form;
8. Weight > 100 kg;
9. Presence of urinary tract stones identified during the current admission (including symptomatic clinical events or incidental findings on baseline abdominal imaging).
10. Known personal or strong family medical history of G6PD deficiency (routine laboratory screening is not required prior to enrollment).

### Intervention

Human pharmacokinetic data indicate a linear relationship between dose and plasma peak concentration. Continuous infusion at higher daily doses is required to sustain plasma levels considered necessary for antioxidant and endothelial effects (19). Our preliminary data revealed a critical dose–response discrepancy: while a dosage of 200 mg/kg effectively improved inflammatory biomarkers in patients with MSAP, no comparable clinical benefit was observed in the SAP cohort (6). This divergence suggests that SAP represents a catastrophic ‘Redox Storm’, where the production of ROS exponentially overwhelms the neutralizing capacity of conventional regimens. Vitamin C exhibits a linear dose-concentration relationship in critically ill patients. Continuous infusion provides stable steady-state plasma concentrations proportional to the daily dose (20). Notably, a comprehensive scoping review has validated the safety of ultra-high-dose intravenous vitamin C, reporting an average well-tolerated dosage of 455 mg/kg/d (ranging from 260 to 925 mg/kg/d) across critically ill and oncological populations (21). Guided by this safety benchmark of 455 mg/kg/d, we have escalated our study dose to 500 mg/kg per 24 h (administered from 06:00 to 06:00 the following day) for 7 consecutive days, aiming to surpass the therapeutic threshold and capture a definitive treatment effect in SAP.

To ensure pharmacological consistency across centers, the study medication is standardized. Vitamin C is sourced from a single manufacturer (Chengdu Brilliant Pharmaceutical Co., Ltd., Chengdu, China; National Medicine Permission Number: H32022732) from one production batch. The medication is supplied in ampoules of 1 g per 5 mL (200 mg/mL). The solvent is water for injection, with sodium metabisulfite, cysteine hydrochloride, disodium edetate, and anhydrous sodium carbonate as excipients. To avoid fluid overload in SAP patients, vitamin C is given as the undiluted original solution. For the intervention group, the weight-based dose (500 mg/kg/day) is drawn into 50 mL light-proof syringes by an unblinded drug preparation nurse (UDPN). The solution is administered continuously via a central venous catheter using a micro-infusion pump at 2 g/h. The control group receives 0.9% normal saline at 10 mL/h to match the vitamin C infusion, for a daily sodium chloride load of approximately 2.16 g. Throughout the intervention period, patients will be closely monitored for adverse effects. If complications such as cystinuria, gout, hyperoxaluria, oxalate deposition, or urate kidney stones occur, the dosage should be adjusted as clinically indicated. In severe cases, the trial may be discontinued.

During the intervention, if an interruption occurs (such as CT transport, infusion pump disconnection, central line failure) the infusion will be temporarily paused and later resumed at the identical constant rate of 2 g/h. Catch-up boluses or rate increases are prohibited. Interruptions under 2 h per 24-h cycle are considered acceptable. Longer interruptions are documented as protocol deviations but the patient remains in the study under ITT analysis.

Vitamin C has low molecular weight and high water solubility, so it is cleared during CRRT. The baseline dose is high enough that plasma levels should stay well above the therapeutic threshold even during continuous renal replacement therapy (CRRT). Keeping the dose unchanged avoids rebound toxicity risk if CRRT is interrupted unexpectedly, and it keeps the protocol consistent across all study centers. We will document CRRT parameters (duration, modality, effluent dose) in Table 1 and include them in statistical adjustments and subgroup analyses to capture how CRRT affects drug exposure.

**Table 1:** Collected procedures, data, and outcomes.

Procedures	Day 0	Day 1	Day 3	Day 7	Day 14	Day 28	Day 180
Eligible criteria	x						
Informed consent	x						
Demographic and medical data	x						
Vital signs	x	x	x	x	x	x	
Laboratory biomarkers^*^	x	x	x	x	x	x	
Plasma vitamin C concentration	x	x	x	x			
SOFA score	x	x	x	x	x	x	
APACHE II score	x	x	x	x	x	x	
Antibiotics							
Surgical intervention							
Continuous renal replacement therapy (modalities, durations, and intensities)	x	x	x	x	x	x	
Mechanical ventilation	x	x	x	x	x	x	
Vasoactive drugs	x	x	x	x	x	x	
Gut microbiota	x	x	x	x	x	x	
Fluid retention^#^							
Abdominal CT scan	x			x	x	x	
Death							x
Hospital stay							x

CT, computed tomography; SOFA, sequential organ failure assessment; APACHE II, acute physiology and chronic health evaluation II.

* Laboratory biomarkers include: blood cell analysis, blood gas analysis, C-reactive protein, procalcitonin, coagulation test, liver function, renal function, NT-proBNP, TnI, cytokines, immunoglobulin, pancreatic enzymes.

# The calculation of fluid retention: intravenous fluids + enteral nutrition − (urine output + drainage volume).

### Outcomes

Table 1 shows the collected procedures, data, and outcomes.

### Primary outcome

This study assesses the effect of HDIVC on organ support-free days (OSFD) within the first 14 days in SAP patients. OSFD refers to the number of days a patient does not require circulatory support (vasoactive agents), respiratory support (mechanical ventilation), or renal support (renal replacement therapy). For patients who die within the 14-day observation period, OSFD is assigned a value of 0 day.

### Secondary outcome

The secondary outcomes are: (1) ICU all-cause mortality, defined as death from any cause from randomization to ICU discharge or transfer. (2) Early SAP inflammatory markers, including C-reactive protein and cytokines IL-6, IL-1, and IL-10. (3) Organ function scores, including SOFA and modified Marshall scores. (4) Fluid retention. (5) Disease severity scores, including APACHE II and BISAP scores. (6) Pancreatic necrosis measured by CTSI scores. (7) Other prognostic indicators including the incidence of infected pancreatic necrosis, ICU length of stay, and hospitalization costs. (8) Plasma vitamin C concentrations before and after treatment. (9) Adverse events related to vitamin C. (10) Gut microbiota composition.

### Sample size

The sample size calculation is based on the primary outcome of OSFD within the first 14 days. Assuming the HDIVC group would gain 2 additional OSFD days compared to the control group (SD 5 days), with a two-sided *α* of 0.05 and power of 0.80, the initial calculation using the formula for two independent sample means required 98 patients per group. To account for non-normal distribution of OSFD, the Pitman asymptotic relative efficiency correction expanded the sample by 15% to 113 per group. Allowing for a 5% dropout rate, the final sample is 238 patients (119 per group). The study plans to recruit 10 patients per month over 24 months. Ethical approval has been gained in participating centers. Centralized online training has been completed to ensure protocol adherence. As of May 30, 2026, the lead center has recruited 34 patients. Participating centers are being activated, which will increase recruitment capacity.

### Randomization and blinding

Participants will be strictly enrolled based on predefined eligibility criteria. Stratified block randomization will be employed by each research center. Patients will be allocated to either the HDIVC group or control group, with allocation concealment maintained throughout. Randomization numbers will be generated by statisticians using SAS 9.4 software, employing block randomization within each stratum to ensure balanced group assignment. Expansion of the sample size may be considered if necessary.

Stratification is performed at three levels: (1) participating center (8 centers), and (2) etiology, categorized as biliary, hypertriglyceridemic, alcoholic or other. Due to our rigorous inclusion criteria, all enrolled patients are diagnosed with SAP. We will not proceed stratification by disease severity. The randomization sequence is generated independently for each stratum combination (8 × 4 = 32 strata) by an independent statistician using SAS 9.4, employing variable block sizes of 4 and 6 (randomly alternated) to ensure allocation concealment and balance within each stratum.

To ensure blinding, study medications—either 50 mL vitamin C solution or matching placebo (0.9% normal saline)—will be prepared in identical, opaque, light-proof containers by UDPN according to the randomization list. Both groups will receive equal volumes (50 mL) via identical administration protocols using the standardized micropump (2 g/h). Crucially, the administration through a central vein minimizes potential sensory cues (such as infusion site irritation) that could arise from pH differences, thereby maintaining the blind for participants, clinical staff, and data analysts. All centers will follow this standardized protocol after thorough training and the study medicine is provided by one center to avoid bias.

The data management and analysis will implement a two-stage unblinding process. After all data have been locked following blinded data verification, the group assignments for each participant will be disclosed for statistical analysis. When the statistical analysis completes, the specific group assignments (intervention group or control group) will be disclosed to assess the study outcomes.

During the treatment period, if complications such as urinary crystals or unexplained acute kidney injury (meeting KDIGO guidelines) occur, emergency unblinding will be performed in accordance with GCP procedures. The attending physician will immediately notify both the site principal investigator and the study-wide principal investigator, who will jointly evaluate the event and determine appropriate clinical management. All such events will be documented and reported to the institutional review board as required.

To reduce the risk of unblinding from pronounced clinical responses, several strategies have been implemented. First, the initiation, titration, and withdrawal of life-sustaining therapies (vasopressors, mechanical ventilation, and CRRT) are protocol-driven. Bedside clinicians follow objective physiological targets and international guidelines rather than subjective judgment. Second, the primary outcome (OSFD within the first 14 days) is a definitive, objective outcome not subject to observer bias. Third, clinical data extraction and calculation of secondary outcomes (SOFA and modified Marshall scores) are performed by independent, blinded research personnel not involved in daily patient management. Investigators will monitor and record events that could lead to unblinding, including unexplained severe AKI or suspected oxalate nephropathy, and spurious glucose readings or glucometer errors.

### Data collection and management

To accurately capture the true severity and clinical heterogeneity of severe acute pancreatitis, comprehensive baseline and dynamic clinical parameters will be systematically recorded in the electronic Case Report Forms (eCRF). These explicitly include: (1) pancreatic enzyme levels over time; (2) daily APACHE II and SOFA scores; (3) the exact degree of extrapancreatic organ involvement and specific organ failures (e.g., respiratory, cardiovascular, and renal impairment); and (4) the requirement and duration of advanced therapeutic interventions, specifically including mechanical respiratory support, hemodynamic support (vasopressors), and CRRT. Recording these decisive clinical variables ensures a rigorous evaluation of the true therapeutic value of HDIVC in the overall care of SAP. Missing data for the primary and secondary outcomes will be addressed using multiple imputation by chained equations (MICE), assuming the data are missing at random. If not, last observation carried forward (LOCF) will be used. Source documents will be maintained for verification. The database will be locked after monitoring and queries resolution for final analysis.

Fecal samples will be collected within 24 h of enrollment (baseline), on treatment Day 7, and on Day 14. Samples will be frozen at −80 °C and shipped to the central laboratory. Total genomic DNA will be extracted, and the V3–V4 hypervariable region of the 16S rRNA gene will be PCR-amplified and sequenced by a certified sequencing service provider.

To minimize inter-center variability, concomitant treatments such as fluid resuscitation and antibiotic use will be guided by the same recommendations for severe acute pancreatitis management (21). Although minor variations reflecting local practice are expected, adherence to a standardized framework will be reinforced through investigator training and regular monitoring. All concomitant therapies will be prospectively recorded, and potential confounding effects will be addressed through statistical adjustment in the final analysis. Center will be included as a random intercept in the primary mixed-effects model to account for clustering of patients within centers.

### Statistical methods

The statistical analysis will follow an intention-to-treat (ITT), per-protocol (PP), and safety population approach. All statistical tests will be two-sided, with a *p*-value < 0.05 considered statistically significant. Baseline characteristics will be summarized using descriptive statistics. For quantitative variables, descriptive statistics will include calculations of mean, standard deviation, median, minimum, maximum, and interquartile range (IQR). For categorical variables, descriptive statistics will report frequencies and percentages for each category.

The primary outcome, OSFD within the first 14 days, will be analyzed using a linear mixed-effects model with center included as a random intercept, allowing quantification and adjustment for between-center variability. Sensitivity analyses will treat center as a fixed effect or omit center effects to assess robustness. A treatment-by-center interaction test will be performed to evaluate heterogeneity of treatment effect across sites. The intraclass correlation coefficient (ICC) from the null model will be reported. To control the familywise type I error rate for secondary outcomes, a hierarchical (fixed-sequence) testing procedure is applied. The pre-specified order is: (1) ICU all-cause mortality, (2) SOFA score at Day 7, (3) C-reactive protein at Day 7, (4) APACHE II score at Day 7, followed by all remaining secondary outcomes. Each outcome is formally tested only if all preceding outcomes in the hierarchy reach statistical significance at the two-sided *α* = 0.05 level. Outcomes below the hierarchical chain—including cytokine levels (IL-6, IL-1, IL-10), BISAP score, CTSI score, ICU length of stay, hospitalization costs, and gut microbiota composition—are designated as exploratory. Their *p*-values will be reported descriptively without formal inferential claims.

Bioinformatics processing will use QIIME2 with DADA2 denoising to generate amplicon sequence variants (ASVs), and taxonomic assignment will be performed against the SILVA database. Alpha diversity (Shannon, Chao1 indices) will be compared between groups using Wilcoxon rank-sum tests. Beta diversity (Bray-Curtis, UniFrac distances) will be visualized by principal coordinate analysis (PCoA) and tested by PERMANOVA. Differential abundance analysis will be conducted using LEfSe (LDA > 2.0) and MaAsLin2. Longitudinal changes will be analyzed using linear mixed-effects models if serial samples are available.

Prespecified subgroup analyses will examine treatment effects by disease severity and baseline characteristics using interaction tests. All analyses will be performed using SAS 9.4, adhering to CONSORT guidelines.

### Interim analysis and data monitoring committee

An interim analysis will be conducted when approximately 50% of participants have completed the primary outcome assessment. The analysis will use O’Brien–Fleming boundaries, with nominal alpha spending to control the overall type I error rate. Predefined statistical thresholds for efficacy and futility will be used to guide recommendations. An independent Data Monitoring Committee (DMC), including experts in pancreatitis, clinical trials, and statistics, will oversee patient safety, trial conduct, and data integrity. The DMC will operate under a formal charter that specifies its responsibilities, decision-making procedures, meeting frequency, reporting of adverse events (Supplementary material 1). The committee will have the authority to recommend continuation, modification, or early termination of the trial based on interim findings. All interim analyses will be conducted by statisticians independent of the study team, and results will remain blinded to investigators until the trial is completed.

### Safety and adverse event reporting

Safety will be assessed by collecting and recording adverse events (AEs), changes in vital signs, laboratory parameters (including blood, urine, and biochemical tests), and systemic reactions during the treatment period.

#### AEs include

(1) presence of urinary crystals.
(2) occurrence of acute kidney injury (AKI) as defined by KDIGO criteria.
(3) clinical or laboratory signs of unexpected hemolytic anemia (e.g., sudden hemoglobin drop combined with elevated indirect bilirubin), specifically to monitor for undetected G6PD deficiency.

#### SAEs include

(1) death,
(2) life-threatening conditions.
(3) prolongation of hospital stay.
(4) persistent or significant disability.
(5) congenital anomalies or birth defects.

#### Treatment discontinuation criteria

Study drug infusion must be discontinued immediately for any of the following events. Emergency unblinding may be performed if deemed necessary by the attending physician:

(1) new-onset AKI meeting KDIGO Stage 2 or 3 (serum creatinine ≥ 2.0 × baseline or urine output < 0.5 mL/kg/h for ≥ 12 h) judged as potentially related to vitamin C-associated oxalate nephropathy.
(2) microscopic urinalysis showing abundant calcium oxalate crystals (≥50 per high-power field) with acute creatinine rise.
(3) clinical or laboratory evidence of hemolytic anemia (sudden hemoglobin drop, elevated indirect bilirubin, hemoglobinuria).
(4) confirmed hypoglycemia (<3.9 mmol/L) after excluding glucometer interference by venous blood gas confirmation.
(5) any SAE judged as possibly or definitely related to the study drug.

Both AEs and SAEs will be evaluated at 8:00 a.m. every day. Investigators are required to promptly assess the aforementioned adverse events. If the investigator determines that an abnormal laboratory result is clinically significant and not part of the clinical condition, it should be recorded as an adverse event and report to the PI of each research site. Once confirmed, the intervention should be discontinued immediately and proper medication should be taken.

### Monitoring

All investigators (physicians, UDPNs, nurses, pharmacists, and data managers) must complete standardized training and pass a competency assessment before center activation. Study medication originates from a single production batch across all eight centers to ensure pharmacological consistency.

Adherence to the infusion protocol is monitored daily. Study nurses record infusion start time, rate, interruptions, and interruption causes in the electronic CRF. Cumulative interruptions <2 h per 24-h cycle are considered acceptable and interruptions ≥2 h are classified as protocol deviations, with the patient retained in the ITT analysis.

Each center’s PI reviews deviations quarterly and submits summary reports to the DMC. If the deviation rate for any procedure exceeds 10% at a given center, targeted retraining is initiated. The DMC reviews cross-center deviation patterns regularly. Systematic inter-center differences trigger focused quality assurance measures.

A combined approach of central statistical monitoring and periodic on-site monitoring is employed. Concomitant therapies (fluid resuscitation, antibiotics, nutritional support) follow standardized SAP management guidelines across all centers, and all such therapies are prospectively recorded for potential statistical adjustment.

### Expected results

We anticipate that the continuous administration of HDIVC at 500 mg/kg/day for 7 days will result in an improvement in the primary outcome of OSFD within the first 14 days among SAP patients compared to the placebo group. HDIVC is expected to effectively neutralize excessive ROS and halt the progression of MODS. We also expect that HDIVC will lead to improvements in ICU all-cause mortality, early SAP inflammatory markers, organ function scores, fluid retention, disease severity scores, pancreatic necrosis, ICU length of stay, and hospitalization costs.

## Discussion

The evidence for IV vitamin C in critical illness has evolved substantially. The CITRIS-ALI trial (*n* = 167) found no reduction in SOFA scores in sepsis-associated ARDS (22), and the LOVIT trial (*n* = 872) reported that vitamin C (50 mg/kg every 6 h for up to 96 h) increased the risk of death or persistent organ dysfunction in adults with sepsis receiving vasopressor therapy (RR 1.21, 95% CI 1.04–1.40) (23). Meta-analyses have been mixed: Liang et al. (24) found no mortality benefit, whereas Lee et al. (25) reported a mortality reduction with monotherapy. These discordant results suggest that vitamin C efficacy depends critically on disease context, dose, timing, method of administration (bolus injection or micropump infusion), and drug types that affect solution pH (ascorbic acid or sodium ascorbate)—none of the existing large trials targeted SAP specifically. The negative results of CITRIS-ALI trial and LOVIT trial may be attributable, in part, to the use of bolus injection and ascorbic acid (pH ~ 5.5–6.5), which can aggravate acidosis in critically ill patients. In the present trial, we administer sodium ascorbate—a pH-neutral formulation (pH ~ 7.0–7.4)—via continuous micropump infusion, which provides stable steady-state concentrations, avoids bolus-related adverse effects, and may improve tolerability and efficacy. The dose (500 mg/kg/d) is substantially higher than those used in LOVIT trial and our prior SAP trial, selected via pharmacokinetic modeling to achieve higher plasma concentrations required for antioxidant and vasopressor-synthesis effects.

HDIVC represents a promising adjunctive therapy for SAP by targeting oxidative stress and systemic inflammation. If proven effective, the study may provide evidence to support future guidelines. However, several challenges still remain. Patient heterogeneity and variability in disease course may influence treatment response. Patients >100 kg are excluded because weight-based dosing in predominantly adipose excess could yield a disproportionately high drug load. The 50 g/d cap is consistent with established safety limits (21), though this limits generalizability to obese SAP patients. Inter-center variability in supportive care is inevitable in a pragmatic eight-center trial. We mitigate this through centralized training, protocol-driven management, including center as a random intercept, and prospectively recording concomitant therapies. Timely enrollment is critical to capture the early inflammatory phase, but in clinical practice, delays in diagnosis or transfer may limit early intervention. To avoid this as far as possible, we set a 7-day cutoff from the onset of the disease during which vitamin C may exert therapeutic effect on SAP. But the 7-day enrollment window requires organ failure documentation by Day 5 to meet the Atlanta SAP criteria, which may delay or preclude enrollment.

## Glossary

ICU: Intensive care unit
SAP: Severe acute pancreatitis
HDIVC: High-dose intravenous vitamin C
AP: Acute pancreatitis
ROS: Reactive oxygen species
SIRS: Systemic inflammatory response syndrome
MODS: Multiple organ dysfunction syndrome
ARDS: Acute respiratory distress syndrome
COVID-19: Coronavirus disease 2019
RNS: Reactive nitrogen species
ACTH: Adrenocorticotropic hormone
NF-κB: Nuclear factor-kappa B
MSAP: Moderately severe acute pancreatitis
CRP: C-reactive protein
OSFD: Organ support-free days
ERCP: Endoscopic retrograde cholangiopancreatography
eGFR: Estimated glomerular filtration rate
AIDS: Acquired Immunodeficiency syndrome
IL-6/IL-1/IL-10: Interleukin-6/interleukin-1/interleukin-10
SOFA: Sequential organ failure assessment
APACHE II: Acute physiology and chronic health evaluation II
BISAP: Bedside index for severity in acute pancreatitis
CTSI: Computed tomography severity index
IPN: Infected pancreatic necrosis
UDPN: Unblinded drug preparation nurse
KDIGO: Kidney disease: improving global outcomes
GCP: Good clinical practice
PI: Principal investigator
IRB: Institutional review board
MICE: Multiple imputation by chained equations
LOCF: Last observation carried forward
ITT: Intention-to-treat
PP: Per-protocol
DMC: Data monitoring committee
AE: Adverse event
SAE: Serious adverse event
NT-proBNP: N-terminal pro-B-type natriuretic peptide
TnI: Troponin I
CT: Computed tomography
