# Supplementary material for: Effect of high-dose intravenous vitamin C on organ support-free days within the first 14 days in severe acute pancreatitis patients: a multicenter, randomized, double-blind, placebo-controlled clinical trial protocol
Source: Front Nutr. 2026 Aug 6;13:1850105. doi: 10.3389/fnut.2026.1850105 (PMC13489861; doi:10.3389/fnut.2026.1850105)
Supplement: Supplementary file 1 [file Table_1.DOCX]

**Supplemental Material 1**

# Data Monitoring Committee (DMC) Charter

**Purpose**

The DMC is established to safeguard the rights, safety, and well-being of study participants, to ensure trial integrity, and to provide independent oversight of safety, efficacy, and trial conduct.

# Composition

The DMC will consist of at least three independent members:

One clinical expert in pancreatitis/critical care.

One expert in clinical trial.

One independent statistician.

Members will be free of financial or scientific conflicts of interest related to the study.

# Responsibilities

The DMC will:

Monitor participant safety, including adverse events (AEs), serious adverse events (SAEs), and key clinical outcomes (e.g., ICU mortality, renal function).

Review interim efficacy and futility data.

Evaluate trial conduct, including protocol adherence, recruitment rate, and data quality.

Recommend continuation, modification, or early termination of the trial based on accumulated evidence.

# Meetings

The primary interim analysis will occur after approximately 50% of participants have completed the primary outcome assessment. Additional meetings may be convened if significant safety concerns arise. All meetings will be held in closed session, independent of the trial investigators.

# Data Review

Interim analyses will be conducted by independent statisticians. Reports will summarize efficacy, safety, and enrollment data. Only the DMC will have access to unblinded interim results. Investigators and trial staff will remain blinded until study completion.

# Decision-Making

Recommendations will be made by consensus or, if necessary, by majority vote. Recommendations will be communicated in writing to the Trial Monitoring Committee, which will review and act upon them, including notifying ethics committees if required.

# Confidentiality

All deliberations, reports, and interim data are strictly confidential. Information will not be disclosed outside the DMC except through official recommendations. Public disclosure will occur only after the trial has been completed.

# Documentation

Detailed minutes will be recorded for each meeting, summarizing discussions, decisions, and recommendations. All documentation will be archived securely according to Good Clinical Practice (GCP) requirements.
