# Supplementary material for: Effect of high-dose intravenous vitamin C on organ support-free days within the first 14 days in severe acute pancreatitis patients: a multicenter, randomized, double-blind, placebo-controlled clinical trial protocol
Source: Front Nutr. 2026 Aug 6;13:1850105. doi: 10.3389/fnut.2026.1850105 (PMC13489861; doi:10.3389/fnut.2026.1850105)
Supplement: Supplementary file 2 [file Table_2.DOCX]

**Supplemental Material 2**

**Participant Consent Form**

Protocol Title: High-Dose Intravenous Vitamin C for Severe Acute Pancreatitis: A Randomized, Multicenter, Double-blinded, Placebo-controlled Clinical Trial Protocol Across Three Tertiary Hospitals in China

Research Institution: Emergency Department, Shanghai Jiao Tong University School of Medicine Affiliated Ruijin Hospital

**Introduction**
Severe acute pancreatitis (SAP) is a life-threatening form of acute pancreatitis with a rising global incidence—estimated at 13–45 cases per 100,000 in Western countries and increasing in China from 0.19% to 0.71% over the past two decades. Despite advances in understanding and management, mortality remains high at 13%–35%.
In the early phase of SAP, oxidative stress and excessive reactive oxygen species (ROS) drive systemic inflammatory response syndrome (SIRS) and subsequent multiple organ dysfunction (MODS). Vitamin C, a key antioxidant, neutralizes ROS, mitigates inflammation, and protects the endothelial barrier. Experimental studies suggest that vitamin C supplementation improves outcomes in animal models, but clinical evidence remains limited due to small sample sizes and suboptimal dosing.
High-dose intravenous vitamin C (HDIVC) can achieve plasma concentrations far exceeding those attainable by oral administration and may improve outcomes in SAP. Our previous single-center trial (ChiCTR1900022022) suggested potential benefits, but larger multicenter studies are needed to confirm efficacy. Therefore, we propose a multicenter, randomized, double-blind, controlled trial to evaluate the efficacy and safety of HDIVC in SAP.

**If I participate in the study, what will I need to do?**
You will be randomly assigned to either the HDIVC group or the control group (1:1 ratio, 194 patients per group). HDIVC 500 mg/kg/24h or normal saline will be administered via central venous infusion for 7 days. We will collect demographic, laboratory, and imaging data. Stool samples (2 g each, twice total) will be collected for metagenomic testing and destroyed after the study. You must provide truthful medical history, report any discomfort, avoid restricted medications or foods, and notify the researcher of participation in any other studies.

**Are there any risks associated with the study?**
Central venous catheter insertion may cause minor pain, bruising, mild dizziness, or rarely infection. All procedures will follow strict aseptic protocols.

**What are the potential benefits of participating in the study?**
Participation may improve treatment or provide valuable information for SAP management, but benefits cannot be guaranteed.

**Are there any costs or compensation for participating in the study?**
Costs: None.
Compensation: None.
The study drug and stool testing are funded by research grants. Routine clinical care costs remain your responsibility.

**What happens if I suffer harm due to participation in the study?**
If injury related to this study occurs, you are entitled to free treatment and/or compensation.

**Will my information be kept confidential?**
Your personal data and biological specimens will be coded and stored securely. Identifiable information will not be disclosed except as required by law or approved by you. Only authorized personnel may access your data. Publications will not reveal your identity.

**Am I required to participate?**
Participation is voluntary. You may withdraw at any time without affecting your medical care or rights. The investigator may terminate your participation for safety reasons or protocol noncompliance.

**Informed Consent Form Signature Page**

I have read this informed consent form.

I have had the opportunity to ask questions and all questions have been solved.

I understand that participation is voluntary.

I can withdraw at any time without penalty.

If I need other treatments, fail to follow the study protocol, experience a study-related injury, or if continued participation may pose increased risk, the investigator may discontinue my participation.

I will receive a copy of this signed form.

Subject Name:
Subject Signature:
Date:

Legal Representative Name:
Legal Representative Signature:
Date:

Witness Name:
Witness Signature:
Date:

(Note: A witness signature is required if the subject is illiterate. A legal representative signature is required if the subject lacks/has limited capacity to consent.)

Investigator Statement:
I have presented this document to the subject, requested that they read it carefully, and answered all questions.
Investigator Name:
Investigator Signature:
Date:
